# Impact of Future Remnant Liver Volume on Post-Hepatectomy Regeneration in Non-Cirrhotic Livers

**DOI:** 10.3389/fsurg.2014.00010

**Published:** 2014-04-21

**Authors:** Duilio Pagano, Salvatore Gruttadauria

**Affiliations:** ^1^Mediterranean Institute for Transplantation and Advanced Specialized Therapies (ISMETT), University of Pittsburgh Medical Center in Italy, Palermo, Italy; ^2^Department of Surgery, University of Pittsburgh, Pittsburgh, PA, USA

**Keywords:** living related liver transplantation, regeneration, liver resection, portal vein embolization

## Abstract

**Objective:** The purpose of the study is to detect if some parameters can be considered as predictors of liver regeneration in two different patient populations composed of in living donors for adult to adult living donor liver transplant and patients with hepatic malignancies within a single institution.

**Summary Background Data:** Preoperative multi-detector computed tomography volumetry is an essential tool to assess the volume of the remnant liver.

**Methods:** A retrospective analysis from an ongoing clinical study on 100 liver resections, between 2004 and 2010. Seventy patients were right lobe living donors for liver transplantation and 30 patients were resected for treatment of tumors. Pre-surgical factors such as age, weight, height, body mass index (BMI), original liver volume, future remnant liver volume (FRLV), spleen volume, liver function tests, creatinine, platelet count, steatosis, portal vein embolization, and number of resected segments were analyzed to evidence potential markers for liver regeneration.

**Results:** Follow-up period did not influence the amount of liver regenerated: the linear regression evidenced that there is no correlation between percentage of liver regeneration and time of follow-up (*p* = 0.88). The pre-surgical variables that resulted markers of liver regeneration include higher preoperative values of BMI (*p* = 0.01), bilirubin (*p* = 0.04), glucose (*p* = 0.05), and gamma-glutamyl transpeptidase (*p* = 0.014); the most important association was revealed regarding the lower FRLV (*p* < 0.0001) and percentage of liver regeneration. The stepwise regression revealed a strong impact of FRLV (*p* < 0.0001) on the other predictor variables.

**Conclusion:** Liver regeneration follows similar pathway in living donor and in patients resected for cancer. Small FRLV tends to regenerate more and faster, confirming that a larger resections may lead to a greater promotion of liver regeneration in patients with optimal conditions in terms of body habitus, preoperative liver function tests, and glucose level.

## Introduction

The main troubleshooting for a successful hepatic resection is closed related to the regenerative properties of the liver in response to a greater tissue excision after resective surgical therapies for primary or secondary tumors of the liver and after living donor liver transplantation. The human liver is able to regenerate due to a hyperplastic reaction in the remnant liver (1). However, a “small-for-size” syndrome can occur when the excised liver parenchyma is mayor of the 80% of the total liver volume and the hepatic function does not sustain physiologic needs (2). Preoperative multi-detector computed tomography (MDCT) volumetry is an essential tool to assess the volume of the liver remnant for surgical success (3–8). An increased interest in the outcomes of major hepatectomy for adult to adult living related liver transplantation (LRLT) has likely contributed to these breakthroughs. Clearly, LRLT represents the natural evolution of other surgical procedures, namely reduced-size liver transplantation and split liver transplantation (4), and is based on the segmental anatomy of the liver and on its peculiar capacity to regenerate. LRLT was initially performed successfully in the pediatric population (5), and then proposed as one of the most effective measures to counteract organ shortage in adults (9–12). However, although surgical techniques of excellence and major improvements in perioperative management are now a reality in referral centers for liver surgery, there are still several issues that make this major surgical procedure extremely worrisome, especially when considering the tragic sequels of post-resection liver failure (13). Although appropriate liver remnant volume after resection ensures the liver’s ability to regenerate, regeneration progresses at variable rates in patients. Preoperative and postoperative MDCT scans have been used as a means to study the effects of perioperative factors such as splanchnic hemodynamics and middle hepatic vein harvesting on liver regeneration (10–12). Nevertheless, few papers have studied pre-surgical clinical and biochemical factors that may influence liver regeneration rate. Some studies show that pre-surgical factors such as age, gender, body mass index (BMI), native liver disease, chemotherapy, platelet count, and steatosis might influence liver regeneration (2, 14–17). The aims of this study were to compare liver regeneration after liver resection in living donors for LRLT and patients with malignancies within a single institution and determine if pre-surgical factors such as age, weight, height, BMI, original liver volume, FRL, spleen volume, liver function tests, creatinine, platelet count, steatosis, portal vein embolization (PVE), and number of segments resected have a significant predictive value for liver regeneration.

## Materials and Methods

### Study population

Our study was approved by the “Istituto Mediterraneo per i Trapianti e Terapie ad Alta Specializzazione” (Is.Me.T.T.) Institutional Research Review Board and patients were selected retrospectively from an ongoing clinical study on liver resection. Between November 2004 and January 2010, all patients without chronic liver disease who underwent liver resection of at least two segments, according to the Couinaud classification (18) with pre- and post-operative abdominal MDCT scans were included. A total of 100 patients were identified with 70 patients who were right lobe living donors for LRLT (Group A) and 30 patients who had resection for treatment of hepatic tumors (Group B). Living donor patients were the control in our study since their liver must be immaculate for transplantation.

As described and published elsewhere, we adopted validated methods for MDCT technique and for volumetric estimations of the liver and the spleen (9).

### Evaluation of pre-surgical factors

Blood biochemical tests were performed prior to operation in all 100 patients. Values drawn from electronic medical records include: alanine aminotransferase (ALT), aspartate aminotransferase (AST), albumin, total bilirubin, gamma-glutamyl transpeptidase (GGT), glucose, platelet count, and PT/INR. Steatosis was measured using the Hounsfield units of the liver from a basal CT scan and using the spleen Hounsfield unit as a reference value. Portal hypertension was measured indirectly by measuring the diameter of the common portal vein on portal venous phase images. We also noted the number of Couinaud segments removed and previous PVE from patients with tumors.

### Type of liver resection in patients with liver malignancies

Couinaud classification was used for defining major liver resections with the excision of three or more liver segments and included Right or Left Hepatectomies, Extended or not. The right hemiliver was removed in all of the patients in Group A, Couinaud segments 5–8. The technical breakthroughs and surgical skills developed in the LRLT were used even for patients with liver tumor. Group B consisted of 15 patients with primary neoplasm (hepatocellular carcinoma in 5 cases, 7 cases of intrahepatic cholangiocarcinoma, in 1 case hilar cholangiocarcinoma, in 1 case gallbladder cancer, and in 1 case huge hepatobiliary cystadenoma), and the 15 remnant with liver metastases (from ileal neuroendocrine tumor in 1 case, from ovarian leiomyosarcoma in 1 case, from gastrointestinal stromal tumor in 1 case, and in 12 cases from colorectal cancer).

### Living related liver donation

As previously described elsewhere, living donor safety has to be the first priority during the entire process of LRLT, from the first day of evaluation through the entire follow-up period (9, 19). In our center a step-by-step work-up protocol for donor evaluation has been designed and scientifically accepted and published in 2007 for ensuring donor safety and, additionally, for confirming that the donor is capable of providing a suitable graft for the recipient (20). “All donors went through a complete evaluation process, managed by a multidisciplinary team consisting of clinical psychologists, hepatologists, anesthesiologists, transplant surgeons, referring physicians, and family doctors. The evaluation process was completed in 3 days, with blood work, ultrasound, and consults on the first day; Volumetric Angio Computed Tomography scan and Cholangio Nuclear Magnetic Resonance Imaging (MRI) on the second day; and liver biopsy on the third day. The operation was performed with a bilateral subcostal incision, with upper midline extension (Mercedes incision). Mobilization of the right liver and skeletonization of the retro-hepatic inferior vena cava with ligation of all accessory hepatic veins was performed using the usual piggy-back technique, the only difference consisting in the preservation of accessory veins larger than 0.8 cm in diameter. Intraoperative cholangiogram was always performed, as was intraoperative ultrasound, to confirm the transection plane, which follows the Cantlie line with no vascular occlusion. Isolation of the right hepatic artery was always performed, while isolation of the right portal vein was performed prior to the parenchymal transection only when feasible. The middle hepatic vein always remained with the donor. The following four sequential techniques were performed for the hepatic parenchymal transection: (1) parenchyma tissue fragmentation and skeletonization of biliary-vascular structures with the ultrasonic dissector or water pressure dissector; (2) vascular hemostasis and biliostasis of the minuscule biliary ducts through the use of micro surgical clips and the radiofrequency dissector; (3) section of fibrous and vascular-biliary structures with electrocautery; and (4) suction of organic and irrigation fluids mixed with parenchymal detritus using the aspirator and the integrated aspirator in the ultrasonic dissector. The setting of the ultrasonic dissector was 90% in amplitude, with high tissue selection, while the irrigation rate was 5 ml/h, with suction set at maximum strength. This was applied after the liver capsule was opened by diathermy, set on coagulation at 70 W. The radiofrequency setting was 75 W, and the irrigation rate was between 2.5 and 5 ml/h. The division of the biliary duct was performed just before the end of the parenchymal transection, and always sharply” (20).

### Statistical analyses

All statistical analyses were performed by using the IBM SPSS Statistics 18 (SPSS Inc., Chicago, IL, USA). Group data were expressed as mean ± SD. Regeneration percentages and pre-surgical factors were compared between living liver donor patients and patients with tumors using the Student’s two-sample *t*-test or the Wilcoxon Rank-Sum test with α = 0.05. The effects of the number of Couinaud segments, PVE, and type of liver disease on percentage of regeneration were analyzed in patients with neoplasms using two-sample *t*-tests. A percentile analysis of percent of regeneration was performed for all patients. Pre-surgical factors were compared between living liver donor patients and patients with malignancies using the Student’s two-sample *t*-test or the Wilcoxon Rank-Sum test with α = 0.05. A multiple regression analysis was performed at least to compare the regeneration parenchymal rate to the future remnant liver volume (FRLV): the regression coefficient of each any analyzed factor was defined as the partial regression coefficient because it represents the contribution to the response comparison after it has been adjusted for the other predictor variables.

## Results

### Living liver donors (Group A) vs. patients with liver tumors (Group B)

Patient characteristics and pre-surgical factors were different between the two groups of cases except for weight, serum bilirubin level, percentage of prothrombin time, and diameter of portal vein. Factors that show significant difference between groups (*p* < 0.05) included: height, BMI, original liver volume, ALT, albumin, AST, creatinine, GGT, platelet, INR, steatosis, spleen volume, and glucose (Table 1). Our study shows that the significant differences in the mean value of some pre-surgical factors examined in this study between the two groups induced a wide variety of distribution of percent liver regeneration. However, the percent regeneration rate did not differ (Wilcoxon Rank-Sum test, *p* = 0.357) between the two groups, being in Group B, 86.38 ± 56.91 (95% Confidence Interval, CI: 64.12, 108.64), and in Group A, 94.68 ± 37.52 (95% CI: 85.73, 103. 62).

**Table 1:** **Demographic characteristics for all patients and comparison of pre-surgical factors between patient types: living liver donors and patients with liver malignancies**.

Pre-surgical factor	Living liver donors (*n* = 70)	Patients with liver malignancies (*n* = 30)	Overall cases (*n* = 100)	*p*-Value
	Mean ± SD	Mean ± SD	Mean ± SD	
Age (years)	32.38 ± 9.05	58.70 ± 14.06	40.28 ± 16.19	0.000^a^
Weight (kg)	69.44 ± 11.18	72.17 ± 12.63	70.26 ± 11.64	0.29
Height (cm)	170.54 ± 8.83	166 ± 9.27	169.18 ± 9.16	0.022^a^
BMI	23.80 ± 2.95	26.16 ± 3.87	24.51 ± 3.42	0.001^a^
Original liver volume (cc)	1571.5 ± 278.71	2013.08 ± 766.36	1703.98 ± 517.25	0.002^a^^,^^b^
Future remnant liver (cc)	584.3 ± 105.18	677.19 ± 333.78	612.17 ± 205.37	0.636^b^
Bilirubin (mg/dl)	0.69 ± 0.38	0.92 ± 1.83	0.76 ± 1.02	0.521
ALT (U/l)	42.50 ± 11.28	60.33 ± 32.52	47.46 ± 21.03	0.010^a^^,^^b^
Albumin (g/dl)	4.25 ± 0.38	3.28 ± 0.73	3.98 ± 0.67	0.000^a^^,^^b^
AST (U/l)	21.01 ± 6.25	77.07 ± 144.12	29.37 ± 2.74	0.000^a^^,^^b^
Creatinine (mg/dl)	0.812 ± 0.18	0.925 ± 0.24	0.844 ± 0.21	0.016^a^
GGT (U/l)	27.87 ± 12.28	181.71 ± 161.73	72.27 ± 111.24	0.000^a^^,^^b^
Platelet (10^3^/μl)	222.47 ± 46.55	271.39 ± 104.47	236.44 ± 71.22	0.033^a^^,^^b^
Prothrombin time (%)	101.87 ± 18.15	93.76 ± 22.04	99.55 ± 19.57	0.064
PT/INR	0.97 ± 0.10	1.07 ± 0.16	1.00 ± 0.13	0.005^a^
Steatosis (Hounsfeld units)	1.17 ± 0.14	1.09 ± 0.17	1.15 ± 0.15	0.023^a^
Portal vein diameter (mm)	12.91 ± 2.22	13.44 ± 2.33	13.08 ± 2.25	0.288
Spleen volume (cc)	260.04 ± 125.57	307.69 ± 125.57	274.74 ± 109.41	0.003^a^^,^^b^
Glucose (mg/dl)	97.1 ± 2.9	75.01 ± 25	88.01 ± 9.01	0.002^a^^,^^c^

*^a^ Statistically significant*.

*^b^ Wilcoxon Rank-Sum test*.

*^c^ Fisher Exact test*.

*All others are *t*-tests*.

*SD, standard deviation*.

### Patients with liver tumors (Group B)

**Figure 1:**
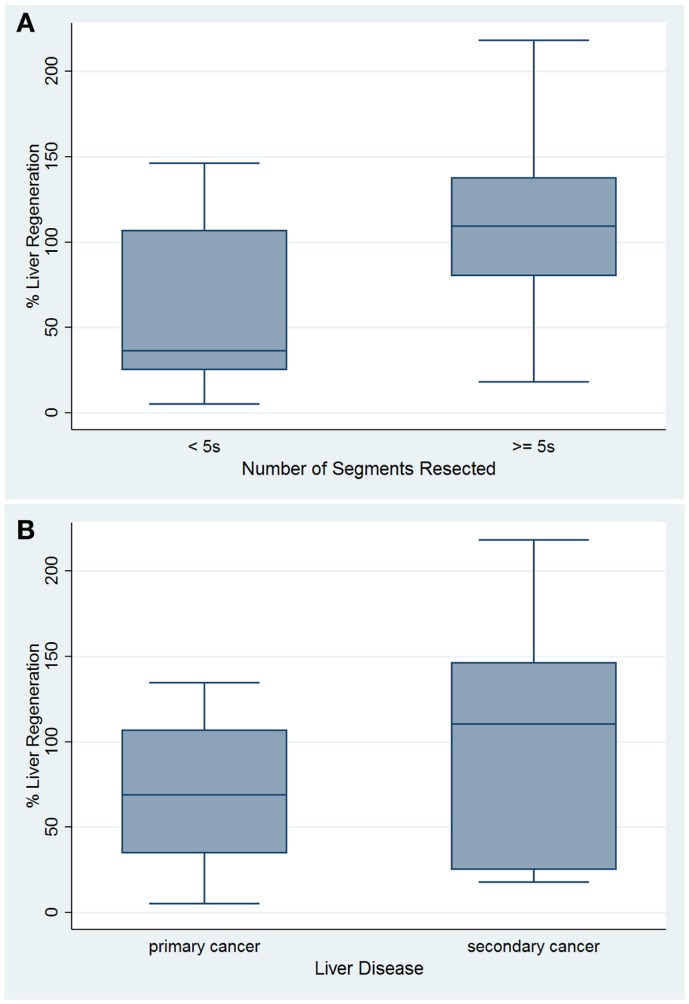
**Box plots of percentage of liver regeneration by number of segments resected (A) and based on type of liver malignancy (B)**.

**Figure 2:**
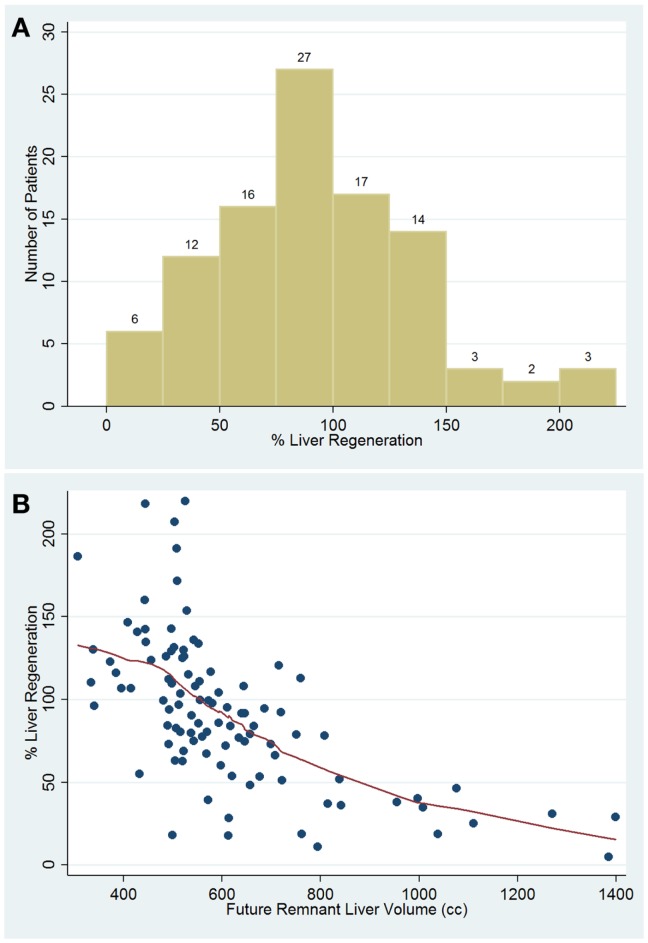
**Percentage distribution of liver regeneration rate in cases of control group and in patient with liver malignancies (A)**. Scatter plot percentage of liver regeneration compared with future remnant liver volume **(B)**.

Analyzing Group B, the plot of percent liver regeneration vs. time of follow-up did not evidence a clear influence with an univariable regression coefficient of −0.040 and a *p*-value of 0.877 (*r*-squared = 0.0009, 95% CI: −0.559, 0.480). Nevertheless, in accord to the number of Couinaud segments resected, we recorded 16 cases who underwent to more/equal five segments resection with an average 112.403 ± 57.859 percentage of liver parenchymal regeneration (95% CI: 81.572, 143.233), in contrast with the remnant 14 patients, 56.643 ± 47.726 (95% CI: 85.73, 103. 62). Clearly, the two-sample *t*-test comparison of the percentage regeneration by number of segments resected evidenced that who had more than five segments resected showed greater liver regeneration (*p* = 0.008) (Figure 1A). In this settings, we compared the percentages of liver regeneration in eight patients affected by liver malignancies who underwent PVE (105.993 ± 49.922, 95% CI: 64.257, 147.728) with the remnant 22 (79.250 ± 62.259, 95% CI: 51.646, 106.854), showing no significant difference (*p* = 0.285). The 15 patients resected for primary liver malignancy evidenced an average of 69.696 ± 10.736 percentage of liver regeneration (95% CI: 46.668, 92.724) (Figure 1B), and when compared with the other 15 operated for liver metastases (103.066 ± 18.319, 95% CI: 63.773, 142.358), no significant difference was detected (*p* = 0.063) (Figure 2A).

### Liver regeneration over/under twentieth percentile analysis

Patients under the twentieth percentile regenerated 50% or less. This was used as a cut-off to separate all 100 patients into other two different groups: optimal regeneration and sub-optimal regeneration (Table 2). Percentile analysis showed that patients above the twentieth percentile regenerated approximately 50% or more according to the FRLV. A significant differences in the mean value of some pre-surgical factors was examined between patients above the twentieth percentile for percent liver regeneration (*n* = 21) and patients below the twentieth percentile for liver regeneration (*n* = 79). Factors that showed significant difference between groups include: BMI, FRLV, GGT, and serum levels of bilirubin and glucose (*t*-test and Wilcoxon Rank-Sum test, *p* < 0.05). (Table 3) The two-sample *t*-test and Wilcoxon Rank-Sum (Mann–Whitney) test of the average FRL for all of the cases under the twentieth percentile (887.754 ± 260.09; 95% CI: 769.35, 1006.14) and for all of the cases over the above percentile cut-off (538.910 ± 101.51; 95% CI: 516.17, 561.64) showed a significant inverse correlation between the percent liver regeneration and FRLV for all patients (*p* < 0.0001) (Figure 2B).

**Table 2:** **Descriptive percentile distribution of percentage liver regeneration for all patients**.

Percentile	Centile (% liver regeneration)	95% Confidence interval
10	31.26	(18.87, 45.04)
20	52.32	(37.04, 68.26)
30	72.32	(53.75, 79.71)
40	80.07	(72.96, 91.74)
50	91.77	(80.20, 99.53)
60	99.58	(91.80, 110.98)
70	111.98	(100.89, 125.43)
80	126.16	(113.65, 136.22)
90	142.64	(130.62, 185.81)
100	219.96	(219.96, 219.96)

**Table 3:** **Comparison of risk factors between patients above (*n* = 21) and below the twentieth percentile (*n* = 79) for and liver regeneration**.

Pre-surgical factors	Under twentieth percentile	Over twentieth percentile	*p*-Value
	Mean ± SD	Mean ± SD	
Age (years)	48.33 ± 21.07	38.13 ± 14.01	0.074^b^
Weight (kg)	72.19 ± 11.51	69.74 ± 11.69	0.394
Height (cm)	165.85 ± 9.35	170.06 ± 8.96	0.061
Body mass index	26.21 ± 3.18	24.06 ± 3.35	0.009^a^
Original liver volume (cc)	1793.19 ± 467.64	1680.25 ± 529.89	0.377
Future remnant liver volume (cc)	887.75 ± 260.09	538.91 ± 101.51	0.000^a^^,^^b^
Bilirubin (mg/dl)	0.96 ± 2.15	0.71 ± 0.41	0.039^a^^,^^b^
ALT (U/l)	52.3 ± 30.21	51.89 ± 53.32	0.650^b^
Albumin (g/dl)	3.72 ± 0.67	4.04 ± 0.66	0.059
AST (U/l)	29.4 ± 17.47	38.97 ± 89.67	0.074
Creatinine (mg/dl)	0.83 ± 0.24	0.84 ± 0.20	0.729
GGT (U/l)	125.45 ± 179.44	58.46 ± 81.50	0.014^a^
Platelet (10^3^/μl)	241.75 ± 59.82	235.08 ± 74.15	0.711
Prothrombin time (%)	101.68 ± 20.58	99.01 ± 19.57	0.588
PT/INR	0.98 ± 0.12	1.01 ± 0.13	0.599
Steatosis (Hounsfield units)	1.11 ± 0.19	1.16 ± 0.14	0.146
Portal vein diameter (cm)	12.68 ± 2.04	13.19 ± 2.31	0.363
Original spleen volume (cc)	246.53 ± 63.85	282.85 ± 118.45	0.411
Glucose (mg/dl)	104.6 ± 33.60	94.19 ± 22.93	0.046^b^

*^a^ Statistically significant*.

*^b^ Wilcoxon Rank-Sum test*.

*All others are *t*-tests*.

### Multiple regression analysis

The analysis included the following predictor variables of liver regeneration: FRLV, BMI, GGT, time of follow-up, spleen volume, albumin levels, steatotic grade, and the number of resected segments, age, and membership in the Group B vs. Group A. The partial regression coefficients evidenced with the stepwise regression revealed a significant association between liver regeneration and FRLV (*p* < 0.0001), BMI (*p* < 0.0001), time of follow-up (*p* < 0.0001), spleen volume (*p* < 0.0001), age (*p* = 0.033), and albumin levels (*p* = 0.038). A unit change in one of these predictive factors, when all other are held constant, contributed to strongly modify the liver regeneration after resection. The magnitude of the change was not dependent on the values at which the other predictor variables were held (Table 4).

**Table 4:** **Multiple regression analysis performed to compare the regeneration parenchymal rate to the future remnant liver volume: the stepwise regression coefficient represents the contribution to the response comparison after it has been adjusted for other predictor variables**.

Pre-surgical factors	Coefficient	*p*-Value	95% Confidence interval
Future remnant liver volume	−0.15	<0.0001	(−0.19, −0.11)
Body mass index	4.06	<0.0001	(1.95, 6.18)
Albumin	−12.8	0.038	(−23.5, −0.67)
Spleen volume	57.7	<0.0001	(29.05, 86.51)
Time of follow-up	0.54	<0.0001	(0.29, 0.79)
Age	−0.54	0.033	(−1.04, −0.04)

## Discussion

Liver resection success relies on the remnant liver’s ability to regenerate. The human liver is able to regenerate due to a hyperplastic reaction in the residual liver (1). Some studies suggest that pre-surgical factors such as age, gender, BMI, native liver disease, chemotherapy, platelet count, and steatosis might have a significant influence on human liver regeneration (2, 14–17). One study in particular demonstrates that an elevated platelet counts in mice after 90% hepatectomy is beneficial for liver regeneration (21). Having seen such correlations in pre clinical setting, we wanted to identify pre-surgical factors as potential marker of liver regeneration in right lobe living donors for living related liver transplant and in patients with liver tumors. There was no statistically significant difference in percent liver regeneration between the two groups, as similarly reported in the study by Zappa et al. regarding the effects on middle hepatic vein harvesting during liver resection on liver regeneration (12). This finding gave us the opportunity to associate the two patient populations together and perform an over/under twentieth percentile analysis for all 100 patients. Looking at patients resected for liver tumors, our study found no correlation between percent liver regeneration and time of follow-up. This suggests that the time after resection alone does not dictate percent liver regeneration and that other factors are at work. Stratification of patients with liver neoplasm by number of Couinaud segments resected showed that patients with five or more Couinaud segments resected had significantly greater percent liver regeneration. Olthoff mentions multiple studies showing the release of pro-inflammatory cytokines such as tumor necrosis factor alpha and interleukin-6 after injury due to resection to initiate the regenerative process (14). Resection of a larger portion of the liver may lead to the release of a greater concentration of these cytokines and promote growth (22, 23). Regarding PVE, although most studies showed significant FRLV growth after this procedure (24–26), in our series there was no significant difference in liver regeneration rate after liver resection when comparing patients who had pre-operative PVE with those who did not. In addition, our analysis also suggests that the type of malignancy, primary vs. metastasis, may have not an effect on liver regeneration in resected patients. The under/over twentieth percentile analysis was performed in all 100 patients since there was no significant difference in percent liver regeneration between Group A and B. The twentieth percentile for percent liver regeneration was used as a cut-off since patients below this value evidenced a percentage of hepatic regeneration inferior of 52.32%, which was considered sub-optimal. There were significant differences in values for the following variables between patients below and above the twentieth percentile: BMI, FRLV, bilirubin, glucose, and GGT. Each of these variables was significantly greater in patients who were below the twentieth percentile for liver regeneration. Our single center study showed that liver regeneration follows similar pathways in living donor of right lobe for living related or unrelated liver transplantation and in patients resected for liver tumors. Finally, the multiple regression analysis was performed on the correlation between percent liver regeneration and FRLV. There is a significant inverse correlation between percent liver regeneration and FLRV. Larger resections may lead to a greater concentration of cytokines and promote growth. However, the key point is to identify the lower limit of FRLV in order to avoid “small-for-size” syndrome and related complications (2, 26, 27).

The most important bias of this study is that the Group B is small and appears to be heterogeneous regarding underlying disease. And for this reason the statistical sub-analysis could be not meaningful due to small study sample size. Otherwise, it should be a primer study for detecting liver regeneration predictors that will be used for other potential comparison between cases with healthy liver parenchyma and patients with liver cirrhosis and portal hypertension. To the best of our knowledge, for the first time in the literature we might observe that for every 100 cc of FRLV in reduction we predicted an average regeneration increase of about 15%. It is the percentage that was adjusted for all other predictors such as BMI, age, time of follow-up, spleen volume, and albumin levels.

## Conflict of Interest Statement

The authors declare that the research was conducted in the absence of any commercial or financial relationships that could be construed as a potential conflict of interest.
